# Transcutaneous auricular vagus nerve stimulation in healthy individuals, stroke, and Parkinson’s disease: a narrative review of safety, parameters, and efficacy

**DOI:** 10.3389/fphys.2025.1693907

**Published:** 2025-10-24

**Authors:** Machiko Matsuoka, Tomofumi Yamaguchi, Toshiyuki Fujiwara

**Affiliations:** ^1^ Department of Physical Therapy, Juntendo University Graduate School of Health Science, Tokyo, Japan; ^2^ Department of Physical Therapy, Human Health Sciences, Graduate School of Medicine, Kyoto University, Kyoto, Japan; ^3^ Department of Physical Therapy, Juntendo University Faculty of Health Science, Tokyo, Japan; ^4^ Department of Rehabilitation Medicine, Juntendo University Graduate School of Medicine, Tokyo, Japan

**Keywords:** noninvasive brain stimulation, taVNS, central nervous system disorder, neuromodulation, rehabilitation

## Abstract

Among the noninvasive electrical stimulation methods, transcutaneous auricular vagus nerve stimulation (taVNS) regulates the activity of various neural networks in the brain and autonomic nervous system and is expected to be applied clinically in many areas, including in patients with central nervous system, psychiatric, and cardiac diseases. Although systematic reviews and meta-analyses have been conducted on safety and efficacy, the variability of stimulation parameters and the lack of consistency in their effects remain significant issues. Therefore, the present study aimed to provide a comprehensive view of the safety, parameters, and efficacy of taVNS by focusing on studies in healthy participants, patients with stroke, and patients with Parkinson’s disease. A literature search was conducted from October 14 to 25 November 2024, using PubMed, Google Scholar, Web of Science, the Cochrane Library, and Scopus. The following search terms were used: “noninvasive VNS or nVNS or noninvasive vagus nerve stimulation,” “transcutaneous vagus nerve stimulation or tVNS,” and “transcutaneous auricular vagus nerve stimulation or taVNS.” In total, 154 papers were included, of which 139 were on healthy participants, nine on patients with stroke, and six on patients with Parkinson’s disease. The safety of taVNS was relatively high. Although minor side effects were reported, no serious adverse events were attributed to taVNS parameters used. taVNS could regulate brain activity, motor and mental functions, and autonomic nervous system activity in patients with stroke and Parkinson’s disease. Modulation of the autonomic nervous system and cortical excitability was also observed in healthy individuals. However, these effects may depend on the stimulation parameters. The lack of reports on safety and the stimulation parameters used was also highlighted. Further validation of parameters and accumulation of evidence regarding the efficacy of taVNS are necessary.

## 1 Introduction

Transcutaneous auricular vagus nerve stimulation (taVNS) is a noninvasive neuromodulation technique that influences autonomic nervous system activity by stimulating the auricular branch of the vagus nerve (ABVN). The afferent fibers of the ABVN enter the vagal trunk and project to the nucleus tractus solitarius (NTS). Activation of the NTS alters brain activity, including that in the locus coeruleus (LC) and raphe nucleus, while affecting the functions of the prefrontal cortex, basal ganglia, and limbic system (Badran et al., 2018a). These effects improve consciousness, motor function, somatosensory function, and mental health in both healthy individuals and patients with neurological disorders (Gao et al., 2023; Dong et al., 2023; Hagen et al., 2014; Colzato et al., 2018a; Colzato et al., 2018b). Although these effects depend on stimulation parameters, such as current intensity, frequency, duration, and on–off time, the influence of these parameters on the outcome remains unclear. Therefore, optimizing taVNS parameters may offer therapeutic benefits to patients with neurological disorders during neurorehabilitation. Unlike earlier systematic reviews and meta-analyses that primarily assessed aggregate efficacy outcomes (Hua et al., 2023; Ramos-Castaneda et al., 2022; Redgrave et al., 2018), this review aimed to explore the potential clinical applications of taVNS in healthy participants, patients with stroke, and patients with Parkinson’s disease (PD), centering on three aspects: safety, stimulation parameters, and efficacy.

In this review, we focus on healthy individuals, stroke, and PD to align mechanistic insights with clinical translation. Healthy participants were included because most mechanistic studies of taVNS have been conducted in this group, providing essential information on stimulation parameters and physiological mechanisms. Healthy cohorts also offer a low-confounder setting to map taVNS stimulation parameters (frequency, duration, intensity, and on-off interval) onto neurophysiological effects (brainstem/cortical activity, electroencephalogram, and autonomic modulation). Stroke and PD then represent canonical motor network disorders in which these mechanisms are hypothesized to support recovery of motor control, gait, and learning (Mondal et al., 2023; Engineer et al., 2019). In addition, taVNS has recently gained attention as a promising adjunct in neurorehabilitation for these conditions, where novel interventions to improve motor and cognitive outcomes are particularly needed (Dolphin et al., 2022; Galvez-Garcia et al., 2024; Maraver et al., 2020; Shi et al., 2023). Additionally, Zhang et al. (2025) provided a comprehensive review of both invasive and non-invasive VNS mechanisms in stroke rehabilitation. They reported that VNS promotes synaptic plasticity, inhibits inflammatory responses, facilitates vascular regeneration, and protects the integrity of the blood–brain barrier, collectively contributing to functional remodeling after ischemic injury. Furthermore, clinical evidence suggests that invasive VNS and taVNS yield significant improvements in upper-limb motor function post-stroke. Previous research on other neuropsychiatric conditions such as depression, epilepsy, and migraine has already been synthesized in systematic reviews and meta-analyses (Lim et al., 2022; Reuter et al., 2019; Wu et al., 2018), whereas the evidence base for stroke and PD remains comparatively limited and heterogeneous. Focusing on these three groups enables harmonized outcome frameworks [e.g., functional magnetic resonance imaging (fMRI), electroencephalogram (EEG), heart rate variability (HRV), task performance, the Fugl–Meyer Assessment of the upper extremity (FMA-UE), gait metrics, Unified Parkinson’s Disease Rating Scale (UPDRS)] and avoids cross-indication heterogeneity that would obscure parameter–effect relationships central to this review. Optimizing taVNS parameters may offer therapeutic benefits to patients with neurological disorders during neurorehabilitation. Given the novelty of taVNS, the current evidence base is still heterogeneous, with relatively few controlled clinical trials available. Therefore, instead of a systematic review, we adopted a narrative review format to provide an overview of existing studies and to outline key challenges for future standardized research.

Regarding safety, the most commonly reported side effects are ear pain, headache, and numbness (Kim et al., 2022). Researchers have reported a low rate of adverse events and no serious events when applying taVNS to healthy individuals as well as patients with stroke, depression, epilepsy, cognitive impairment, and migraine (Redgrave et al., 2018; Tan et al., 2023; Wang et al., 2022; Wu et al., 2020). No differences in the risk of adverse or serious events were observed between the active and sham stimulation conditions. However, the incidence and impacts of adverse events vary despite the application of the same parameters (Gerges et al., 2024b). Moreover, the relationship between taVNS parameters and the incidence or severity of adverse events remains unknown owing to inconsistent reporting of adverse events. Therefore, the association between these parameters and adverse events should be investigated.

The taVNS parameters vary according to individual stimulation sensitivity and disease specificity. To effectively apply taVNS, establishing stimulation parameters that reflect participant characteristics is essential.

Furthermore, the outcomes used to evaluate the effect of taVNS differ across studies, complicating comparisons and potentially hindering clinical applications (Badran et al., 2018b). This review serves as an initial step in exploring the variability and efficacy of taVNS parameters by focusing on the parameters used for each disease and the associated evaluation indices.

Previous studies have reported the effect of taVNS on neural brain networks and motor performance in healthy individuals and those with stroke and PD (Li et al., 2022; Sigurdsson et al., 2021; Wang et al., 2021). However, a functional magnetic resonance imaging (fMRI) study indicated that varying the taVNS settings, such as frequency and stimulus duration, led to different brain network activities. In addition, HRV, which reflects the function of the autonomic nervous system, changes depending on the stimulus intensity and frequency of taVNS (Badran et al., 2018b; Clancy et al., 2014). Thus, it remains unclear which parameters are most effective in specific settings and populations, including healthy individuals and those with neurological disorders (Thompson et al., 2021). Further studies are required to explore the differences and individual variabilities in the effect of taVNS according to different parameters to optimize and maximize its effect (Yokota et al., 2022; Ng et al., 2024).

## 2 Methods

A literature search was conducted from October 14 to 25 November 2024, using PubMed, Google Scholar, Web of Science, the Cochrane Library, and Scopus. The search terms and Boolean combinations used were: (“Non-invasive VNS” OR “nVNS” OR “Non-invasive vagus nerve stimulation”), (“Transcutaneous vagus nerve stimulation” OR “tVNS”), (“Transcutaneous auricular vagus nerve stimulation” OR “taVNS”).

The target population was defined to include healthy adults, patients with stroke, and patients with PD. This selection was guided by (i) original articles published in peer-reviewed journals and written in English; (ii) the density of parameterized taVNS studies in healthy cohorts; (iii) the neurorehabilitation relevance of stroke and PD, where motor and cognitive endpoints allow cross-study comparability; and (iv) the need to minimize heterogeneity from conditions with disparate pathophysiology and outcomes (e.g., mood or cardiovascular indications), which would compromise our parameter-focused synthesis. For patient populations, all studies involving stroke or PD were eligible, irrespective of study design. Studies were included if they applied taVNS with reported stimulation parameters and outcome measures. Exclusion criteria were: (i) unspecified taVNS parameters, (ii) absence of assessment items, and (iii) preprints and study protocols.

The search terms “tVNS” and “nVNS” may also retrieve studies using percutaneous cervical vagus nerve stimulation (tcVNS, e.g., gammaCore), because using “tVNS” alone could exclude papers that mentioned the auricular approach but were labeled differently. Therefore, all abstracts and full texts identified by our search terms were screened, and non-auricular approaches were excluded. This review includes only studies using auricular stimulation (taVNS). Although transcutaneous cervical vagus nerve stimulation (tcVNS) is also a promising approach, it differs in stimulation site and mechanisms; thus, our scope was limited to taVNS.

Studies involving healthy adults of any age were eligible. No specific age restrictions were applied; however, most included studies were conducted in young to middle-aged adults, with only a few explicitly targeting older adults.

This review was conducted in a narrative format. A systematic literature search was performed, and findings were synthesized qualitatively. No statistical pooling or meta-analysis was undertaken because of the heterogeneity of study designs, stimulation protocols, and outcome measures. Outcomes were extracted and narratively organized into three domains: safety, stimulation parameters, and efficacy. This narrative review was not pre-registered on OSF or any other public repository. Screening and data extraction were performed by a single reviewer.

## 3 Results

### 3.1 Overview

**FIGURE 1:**
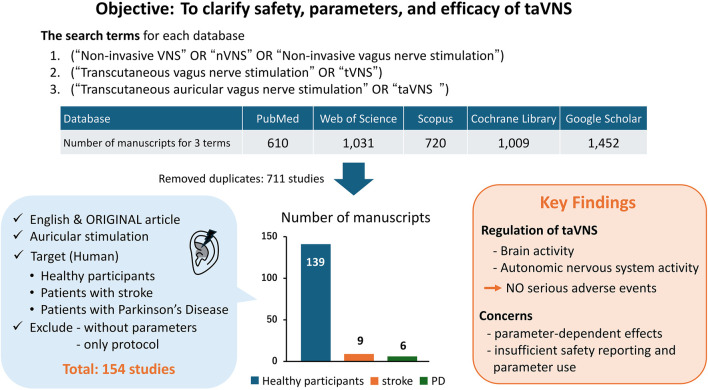
Summary of the present review.

A total of 4,822 records were retrieved from PubMed, Web of Science, Scopus, Google Scholar, and the Cochrane Library. After removing duplicates, 711 unique records remained. Following title/abstract and full-text screening based on the inclusion and exclusion criteria, 154 studies were included in this review (139 healthy, nine stroke, six PD). A simplified flow summary is presented in Figure 1.

Regarding the safety of taVNS, most studies found that the participants did not experience any adverse events. Common side effects included warmth, vibration, and numbness; however, these were not serious. Some studies lacked descriptions of side effects or adverse events.

The parameters varied according to disease and stimulation target, demonstrating less consistency across studies involving healthy participants, patients with stroke, and patients with PD (Tables 1, 2). For each parameter, a frequency of 25 Hz and a pulse duration of 200–300 μs were the most commonly used in both healthy participants and patient populations in the included studies. The most common stimulation intensity was below the pain threshold, followed by the sensory threshold, mild tingling, uniform intensity, and 200% sensory threshold. Methods for determining intensity varied across studies, and there were also nuances in the interpretations of terms such as “tolerable” and “below the pain threshold.” For the on-off interval, a 30-s “on” and 30-s “off” pattern was applied in both healthy participants and patients with stroke; however, the duty cycle in patients with PD varied across studies, including patterns such as 60-s “on” and 10-s “off” or 60-s “on” and 30-s “off”. Some studies applied continuous stimulation without on-off cycle. taVNS was applied for ≤60 min in most studies. However, in healthy individuals, large variations were observed, such as >60 min, only a few minutes, and occasionally during task performance. The outcome measures primarily included disease-specific rating scales and taVNS efficacy measures.

**TABLE 1:** Electrode placement for taVNS (the number of manuscripts).

Category		Healthy participants	Patients with stroke	Patients with Parkinson’s disease
Right or left	Right	2	0	0
	Left	127	8	6
	Bilateral	10	1	0

**TABLE 2:** Electrode attachment sites used in taVNS studies (number of manuscripts by participant type).

Category		Healthy participants	Patients with stroke	Patients with Parkinson’s disease
Target (active)	Tragus	24	2	2
	Cymba concha	90	6	4
	Concha	7	1	0
	Others	20	0	0
Target (sham)	Ear lobe	97	2	5
	Same as active	24	6	0
	Others	12	1	1

Regarding electrode lateralization, both right-sided and left-sided taVNS were found to equally enhance cortical excitability and induce neurophysiological changes in the frontal area (Konakoglu et al., 2023; Camargo et al., 2024). Bilateral stimulation also showed a modulating effect on heart rate variability (Percin et al., 2024; De Couck et al., 2017). Hatik et al. (2023) and Laqua et al. (2014) reported that bilateral stimulation resulted in significantly less stimulation-related pain. Peng et al. (2023) examined the effects of taVNS delivered to different ear targets relative to the lesion (ipsilesional vs. contralesional vs. bilateral vs. sham) in patients with stroke and found that ipsilesional stimulation produced the largest direct brain activation. However, in healthy individuals, activity increased in the wrist extensor muscles on the side opposite to the stimulated side (Konakoglu et al., 2023). Overall, this review found no studies reporting negative results for bilateral stimulation; however, effects may differ between healthy individuals and patient populations, necessitating further investigation. Only 2 studies performed right-sided taVNS (Gauthey et al., 2020; Sinkovec et al., 2021).

Across all included studies (including overlaps), 52 assessed autonomic outcomes (e.g., HR, HRV, pupil size, salivary markers), 60 investigated cortical/neurophysiological indices (e.g., fMRI, EEG, TMS), 42 reported motor outcomes, and 25 assessed cognitive or affective measures. Representative findings included HRV increases and improved pupil size in autonomic measures, modulation of limbic and prefrontal activity in neurophysiological studies, improvements in gait and upper-limb function in motor outcomes, and enhanced attention and reduced anxiety in cognitive/affective measures. Studies involving patients with stroke and those with PD more often evaluated motor function than autonomic nervous system activity. In contrast, studies in healthy participants primarily investigated the neurological and physiological mechanisms of taVNS, employing fMRI, HRV, and electroencephalography (EEG) (Badran et al., 2018a; Gianlorenco et al., 2024; Keute et al., 2021; Rufener et al., 2018; Rufener et al., 2023; Sclocco et al., 2019). Other studies have demonstrated improved performance in tasks involving rewards, fatigue, and memory. In studies on patients with stroke, taVNS contributed to reducing inflammation and vasospasm and enhancing motor and sensory functions, walking speed, gait cycle, balance, and emotional responses (Capone et al., 2017; Chang et al., 2021; Li et al., 2022; Sellaro et al., 2015b; Wang M. H. et al., 2024). Modulation of the default mode network using taVNS underlies these effects (Peng et al., 2023). Regarding the effect of taVNS in patients with PD, the improvement in walking velocity or stride length, anxiety, and modulation of brain areas, such as the superior parietal lobule, anterior central gyrus, posterior central gyrus, middle occipital gyrus, and cuneus, has been reported (van Midden et al., 2024b; Fu et al., 2024; Zhang et al., 2024).

### 3.2 Healthy participants

#### 3.2.1 Safety

Of the 139 studies in healthy participants, 76 (55%) did not report adverse events, 41 (29%) explicitly stated that no adverse events occurred, and 13 (9%) described minor adverse events (e.g., headache, ear pain, tingling, transient fatigue), and nine (6.5%) provided insufficient detail for classification. All reported events were mild and self-limiting, with no serious adverse events documented. Nine studies included sham stimulation, and none observed significant differences in adverse event rates between active and sham groups. 13 studies reported various side effects, including headache; pain; discomfort; nausea; muscle contractions, tingling, or burning sensations; and fatigue. These studies also noted that such side effects were not serious (D’Agostini et al., 2021; Jacobs et al., 2015; Tona et al., 2020). One study concluded that the physiological and potential side effects of taVNS were unknown when used bilaterally because the parasympathetic nervous system of the heart is innervated by the right vagus nerve (Kraus et al., 2007). However, Hatik et al. (2023) subsequently reported that taVNS did not cause bradycardia or hypotension even with bilateral stimulation. Overall, this high rate of non-reporting underscores the potential for underreporting bias; therefore, it was unclear whether any adverse events had occurred. These findings suggest that taVNS is generally safe, although underreporting remains a limitation.

#### 3.2.2 Parameters

**FIGURE 2:**
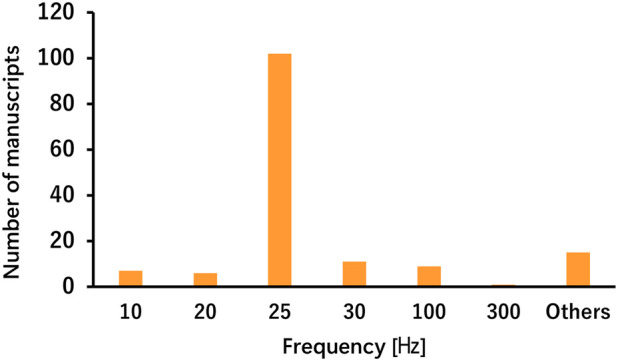
Number of manuscripts by stimulation frequency.

**FIGURE 3:**
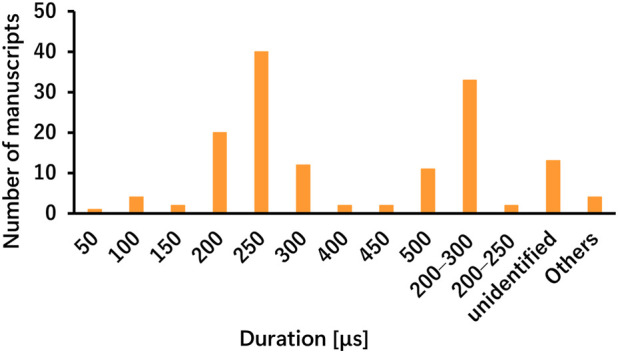
Number of manuscripts by stimulation duration.

The taVNS parameters are summarized in Tables 3a–c as well as illustrated in Figures 2, 3. Table 3 presents the total number of studies that examined multiple frequencies, durations, and intensities. The most commonly used parameters were a frequency of 25 Hz, stimulus interval of 250 μs, and intensity below the pain threshold. However, the stimulus parameters varied among studies. Several studies examined the effects of these parameters. For instance, a study that applied taVNS at different intensities and examined its effects reported that pupillary dilation was most effectively induced at 2 mA (Capone et al., 2021). Regarding other parameters, 500 μs and 10 Hz had the most substantial effect on heart rate (HR) (Badran et al., 2018b), and taVNS at 250 μs, 100 Hz, and 3.0 mA was effective in suppressing pain (St Pierre and Shinohara, 2023; Yokota et al., 2024). Additionally, taVNS at 100 Hz increased cerebellar brain inhibition to a greater extent than that at 25 Hz (van Midden et al., 2024a).

**TABLE 3:** Stimulation parameters in healthy participants (including multiple applications).

a. Frequency	
Frequency [Hz]	Number of published manuscripts
10	7
20	6
25	100
30	11
100	9
300	1
Others	15
Median: 25 Hz, IQR: 0	

b. Duration	
Duration [µs]	Number of published manuscripts
50	1
100	4
150	2
200	20
250	38
300	12
400	2
450	2
500	11
200–300	33
200–250	2
Unidentified	13
Others	4
Median: 250 ㎲, IQR: 100 (200–300)	

c. Current intensity	
Current intensity [mA]	Number of manuscripts
Perceptual threshold	28
200% of the participant’s perceptual threshold	3
Mild tingling (not pain)	19
Below the pain threshold	51
Consistent intensity	36
Not described	2
Others	2

Regarding the location of the electrodes, stimulation of the cymba conchae elicited a stronger and more significant activation of the NTS and LC, key brainstem targets of vagal afferents, compared with stimulation of other auricular sites (Forte et al., 2022; Yakunina et al., 2017). However, the fossa triangularis was also an effective stimulation site (Machetanz et al., 2021), and some studies applied bilateral stimulation (Camargo et al., 2024; Ferstl et al., 2022; Gianlorenco et al., 2024; Hatik et al., 2023; Konakoglu et al., 2023; Laqua et al., 2014; Machetanz et al., 2021; Müller et al., 2021; Ng et al., 2024; Percin et al., 2024). On the other hand, Borges et al. (2020) have reported the cardiac vagal activity may be similarly influenced by afferent vagal stimuli triggered by active and sham stimulation with different stimulation intensities.

Overall, the taVNS parameters are an important consideration; however, the optimal settings remain uncertain because of the variability in parameter combinations and assessment methods.

#### 3.2.3 Effects

The effects of taVNS were predominantly categorized into three domains: modulation of activity in the brainstem and cortical areas; regulation of the autonomic nervous system, including the cardiovascular and gastrointestinal systems; and enhancement of motor and cognitive functions.

##### 3.2.3.1 Modulation of activity in the brainstem and cortical areas

fMRI studies investigating the neuromodulatory effects of taVNS consistently demonstrated increased BOLD signals in key regions, such as the insular cortex, thalamus, prefrontal cortex, and cingulate cortex (Borgmann et al., 2021; Kraus et al., 2007). Furthermore, brainstem regions, including the NTS and LC, were activated following taVNS (Sclocco et al., 2019). These areas constitute the core components of vagal afferent pathways and are critically involved in interoception, autonomic regulation, and emotional processing (Alicart et al., 2021; Dietrich et al., 2008; Johnson and Steenbergen, 2022; Mao et al., 2022). Several studies examining the effects of taVNS on the resting-state brain network have reported increased activation in limbic system regions, including the putamen, caudate, posterior cingulate cortex, amygdala, and parahippocampal gyrus (Keatch et al., 2022; Peng et al., 2018). Teckentrup et al. (2021) reported that taVNS increased network activation in the NTS. However, Kraus et al. (2007) reported that taVNS led to decreased network activation in the limbic system. This apparent discrepancy is likely attributable to variations in stimulation parameters and electrode placement. Specifically, studies reporting increased limbic system activation used electrodes affixed to the cymba concha, whereas those observing reduced activity used electrodes placed on the tragus. Several EEG-based studies demonstrated that taVNS enhanced attention, motor learning, and emotion regulation, indicating its potential as a tool for cognitive and affective modulation (Muller et al., 2022; Wang et al., 2023). Specifically, taVNS modulated alpha and beta brainwave activities, particularly within regions associated with attention, working memory, and cognitive processing (Konjusha et al., 2023; Sun et al., 2021; Vertanen, 2023). Notably, reductions in high-frequency (HF) alpha activity correlated with increased reaction times (RTs), suggesting enhanced cortical alertness and attention (Chen, et al., 2023a; Konakoglu et al., 2023; Konjusha et al., 2022). Changes in motor-related cortical potentials and RT following taVNS suggested enhanced neural efficiency during motor execution (Chen et al., 2024a; Chen et al., 2022; Chen, et al., 2023b; Gadeyne et al., 2022; Gurtubay et al., 2023). Functional connectivity analyses further highlighted alterations in the parietal lobe, reinforcing its role in sensorimotor integration (Poppa et al., 2022; Warren et al., 2020). Emotion-related event-related potentials were also affected by taVNS, with evidence suggesting that taVNS modulated neurophysiological activity in the frontal lobe. Specifically, taVNS enhanced the lateralization of alpha waves toward the right frontal hemisphere during Go/No-Go tasks, potentially supporting improved executive control (Keute et al., 2018; Kuhnel et al., 2020). However, although taVNS consistently produces neurophysiological effects, certain anticipated outcomes, such as alpha suppression, emotional memory formation, emotional responses, and autonomic modulation—have not been reliably replicated. These inconsistencies suggest that the precise mechanisms underlying the effects of taVNS remain poorly understood and warrant further investigation (Camargo et al., 2024). Moreover, motor-evoked potentials (MEPs) and TMS-evoked EEG potentials (TEPs) were used to evaluate the effects of taVNS, particularly on motor-related brain activities. MEPs and TEPs were not significantly influenced at the group level; taVNS with a higher intensity (>2.5 mA) decreased cortical excitability, as evidenced by increased resting motor thresholds, reduced MEP amplitudes, and EEG analysis (D’Agostini, 2023; Pihlaja et al., 2020; Sharon et al., 2021; Ventura-Bort et al., 2021; Warren et al., 2019). taVNS selectively influenced motor performance through the modulation of the GABAergic system in the motor cortex without cholinergic circuits. These were evaluated using short-interval intracortical inhibition and short-latency afferent inhibition. These outcome measures are integral to cognitive processing and motor function (Capone et al., 2015; Horinouchi et al., 2024; Mertens et al., 2022; van Midden et al., 2024a; Wang et al., 2022).

Overall, taVNS modulates cortical activity related to motor function, and these effects can be evaluated using noninvasive brain imaging techniques.

##### 3.2.3.2 Regulation of the autonomic nervous system

Autonomic nervous system effects were assessed using HR, blood pressure, HRV, pupil size, and salivary biomarkers as key outcome measures. Sinkovec et al. (2021) reported significant reductions in HR, left ventricular contractility, and left ventricular output with right taVNS, resulting in a beneficial reduction in left ventricular workload. HRV serves as a robust indicator of parasympathetic activity and sympathetic suppression, with increases in the root mean square of successive differences and HF components of HRV, along with decreases in the low-frequency (LF) to HF (LF/HF) ratio, representing significant markers of autonomic modulation (Geng D. et al., 2022; Geng D. Y. et al., 2022; Petersen et al., 2024; Sclocco et al., 2020; Tobaldini et al., 2019; Toschi et al., 2023). Changes in HRV have been associated with elevated nociceptive withdrawal reflex thresholds and attenuated stress responses, highlighting their potential for treating chronic pain and psychiatric disorders (Yokota et al., 2024). Furthermore, HRV enhancement has been implicated in the restoration of autonomic balance through sympathetic inhibition and parasympathetic activation, particularly in conditions characterized by sympathetic overactivity, such as heart failure (Clancy et al., 2014). Additionally, taVNS improves cardiac baroreflex sensitivity and autonomic modulation (Antonino et al., 2017). HRV modulation is also associated with the mitigation of initial stress responses, nociceptive processing, and the enhancement of cognitive function, highlighting its broader physiological relevance (Austelle et al., 2024; Le Roy et al., 2023). taVNS influences pupil diameter under specific conditions, with significantly greater dilation observed during scotopic illumination and phasic stimulation compared with sham conditions (Skora et al., 2024). The pupillary response appears to scale with the intensity of the taVNS, suggesting possible engagement of the LC–noradrenergic (LC–NA) system (D’Agostini et al., 2023). However, interindividual variability and dependence on prestimulation pupil diameter, a proxy for tonic LC–NA activity, pose challenges to consistency. Further investigations are warranted to determine whether the evoked pupillary changes reflect orienting responses or are attributable to somatosensory perception. Hence, although pupillometry shows promise as a noninvasive biomarker of taVNS effect, its reliability and specificity require rigorous validation (Keute et al., 2019b; Villani et al., 2022; Wienke et al., 2023).

Salivary alpha-amylase (sAA) has been used as a biomarker to evaluate the effects of taVNS, potentially reflecting its NA activity. Although some studies have reported significant increases in sAA following taVNS, others have found no difference compared with sham conditions, resulting in inconsistent findings. Although sAA elevation has been observed under both sustained and brief stimulations, it remains unclear whether these changes directly correspond to NA activation. However, further studies are required to validate the reliability of sAA as a biomarker. Overall, although sAA has potential as an indicator of the effects of taVNS, further validation studies are required (Bömmer et al., 2024; D’Agostini et al., 2022; Fischer et al., 2018; Zhu et al., 2022).

Furthermore, in a study that simultaneously evaluated neurological indices, such as the autonomic nervous index and EEG, pupil dilation after taVNS suggested temporary noradrenaline activation and was associated with autonomic nervous regulation; however, no evident effect was observed on EEG (Lloyd et al., 2023). Thus, although effects have been observed for a single endpoint, inconsistent results have been reported across endpoints. In terms of gastrointestinal effects, HF taVNS enhances gastric motility by increasing the amplitude of peristaltic waves (Frokjaer et al., 2016; Steidel et al., 2021; Teckentrup et al., 2020). Furthermore, taVNS strengthens gastric-to-brain connectivity in the NTS and midbrain while simultaneously enhancing connectivity across broader brain regions (Muller et al., 2022). However, Gancheva et al. (2018) reported that taVNS did not acutely modulate the autonomic tone to the visceral organs, indicating discrepancies in findings between studies.

##### 3.2.3.3 Enhancement of motor and cognitive functions

To investigate the effects of taVNS on the performance, Hatik et al. (2023) examined the effects of taVNS on exercise-induced pain and fatigue. Although taVNS significantly reduced pain and fatigue, it did not improve exercise performance. The observed fatigue recovery effect was attributed to attenuation of exercise-induced sympathetic overactivity by taVNS. Performance outcomes appear to be strongly influenced by individual motivation during experimental tasks (St Pierre and Shinohara, 2023; Wang et al., 2022).

Combining taVNS with interference control training enhances both cognitive function and task performance (Borges et al., 2019). In contrast, failure to apply taVNS at a certain time may impair motor learning (St Pierre and Shinohara, 2023). Studies investigating the effects of taVNS on memory have reported improved memory task scores (Cibulcova et al., 2024; Giraudier et al., 2020; Muller et al., 2022; Yakunina et al., 2017), although no significant effects have been observed on delayed recall (Keatch et al., 2022). Taking advantage of the overlapping brain modulation regions of taVNS and transcranial direct current stimulation (tDCS), a combined stimulation protocol yielded greater enhancement in working memory performance than either intervention alone (Zhao et al., 2022). Furthermore, taVNS may modulate selective aspects of memory processing.

In a multiday fear conditioning and extinction paradigm, Szeska et al. (2020) demonstrated that long-term use of taVNS enhanced the amygdala modulation of the fear-enhancing startle and its cognitive effects. Administering taVNS during the memory extinction phase facilitates the suppression of fear-enhancing startle responses and cognitive risk assessment, thereby reducing the likelihood of fear reinstatement. Collectively, these findings suggest that taVNS improves cognitive and memory functions and may influence memory processing mechanisms, potentially contributing to enhanced learning.

Based on the modulatory effects of taVNS on emotion and mood, studies have suggested that taVNS reduces emotional reactions or anxiety (Burger et al., 2016; Burger et al., 2017 Burger et al., 2018; Burger, 2019; Burger et al., 2019; Ferreira et al., 2024; Finisguerra et al., 2019; Jongkees et al., 2018; Keatch et al., 2023; Sellaro et al., 2018; Steenbergen et al., 2021; Szeska et al., 2021) and improves positive mood during effort tasks (Ferstl et al., 2022). Specifically, the lower the baseline positive mood, the more immediate was the improvement in motivation with taVNS. Regarding its effects on the reward system, some studies have reported the activation of reward functions, indicating increased motivation for reward acquisition rather than effort maintenance (Ferstl et al., 2022; Neuser et al., 2020). Conversely, taVNS does not result in reward activation, regardless of the reward type, scale, or trial difficulty in effort tasks (Lucchi et al., 2024). Neurological investigations have shown that taVNS may enhance the inhibitory control of emotions by acting within the inhibitory control network of the prefrontal cortex (Zhu et al., 2023).

Several studies have demonstrated the effectiveness of taVNS in addressing obesity, sleep disorders, pain, motion sickness, and language skills (Alicart et al., 2021; Altinkaya et al., 2023; Anzolin et al., 2023; Crupper, 2023; Kozorosky et al., 2022; Honda et al., 2024; Jackowska et al., 2022; Llanos et al., 2020; Maharjan, 2018; Mertens et al., 2020; Molefi et al., 2023; Müller et al., 2021; Obst et al., 2020; Phillips et al., 2021; Thakkar et al., 2020; Vosseler et al., 2020; Vishal et al., 2025).

### 3.3 Stroke

#### 3.3.1 Safety

No serious adverse events were observed during the experiments in six of the nine studies. Li et al. (2022) noted that 2 of the 60 participants experienced skin redness, which promptly and completely subsided after adjusting the current intensity. Capone et al. (2017) reported no adverse events or discomfort. Two studies did not describe adverse events (Baig et al., 2019; Peng et al., 2023).

#### 3.3.2 Parameters

All nine studies provided detailed information on the stimulation parameters (Baig et al., 2019; Capone et al., 2017; Chang et al., 2021; Huguenard et al., 2024; Li et al., 2022; Liu et al., 2024; Peng et al., 2023; Wang L. et al., 2024; Wang M. H. et al., 2024). The parameters are summarized in Tables 4a–c. The predominant frequencies used were 25 Hz, with a duration of 300 µs and a duty cycle of 30 s “on” and 30 s “off”. However, there were inconsistencies in determining the intensity across studies. Most studies implemented session schedules lasting ≥10 days, indicating medium-to long-term intervention strategies (Capone et al., 2017; Chang et al., 2021; Li et al., 2022; Liu et al., 2024; Wang M. H. et al., 2024).

**TABLE 4:** Stimulation parameters in patients with stroke.

a. Frequency	
Frequency [Hz]	Number of published manuscripts
20	4
25	4
30	1
Median: 25 Hz, IQR: 5 (20–25)	

b. Duration	
Duration [µs]	Number of published manuscripts
100	1
250	1
300	5
500	2
Median: 300 ㎲, IQR: 0	

c. Current intensity	
Current intensity [mA]	Number of published manuscripts
below the pain threshold	2
tolerable level	3
200% Perceptual Threshold	1
discomfort level	1
uniformly determined	1
Not defined	1

#### 3.3.3 Efficacy

The clinical efficacy of taVNS in conjunction with rehabilitation interventions and other neuromodulatory devices has been demonstrated. Of the nine stroke studies, three applied taVNS alone and reported improvements in upper-limb motor recovery, sensory function, or depressive symptoms. The remaining six studies combined taVNS with rehabilitation training, robotic therapy, or tDCS, reported enhanced functional outcomes such as gait, balance, and activities of daily living (Baig et al., 2019; Capone et al., 2017; Chang et al., 2021; Li et al., 2022; Wang L. et al., 2024; Wang M. H. et al., 2024). In the robotic therapy trials, participants received identical robotic training with either taVNS or sham taVNS; both studies reported larger gains in upper-limb function in the taVNS + robotic arm relative to robotic + sham, indicating an added benefit of taVNS under matched co-intervention (Capone et al., 2017; Chang et al., 2021). In trials combining taVNS and tDCS, arm structures varied (e.g., taVNS + tDCS vs. tDCS alone vs. taVNS alone vs. control). The combination arm generally showed the largest improvements; however, formal taVNS × tDCS interaction tests were not reported, so synergy cannot be established. Across studies, taVNS-alone arms improved motor outcomes versus their control/sham comparators, albeit with small samples and heterogeneous dosing (Wang M. H. et al., 2024). Futhermore, combining taVNS with rehabilitation has demonstrated notable improvements in motor and sensory functions and in managing post-stroke depression (Baig et al., 2019; Li et al., 2022). Peng et al. (2023) indicated that all active taVNS conditions (ipsilesional, contralesional, and bilateral) significantly attenuated activity in the contralesional default mode network compared with the sham condition. However, only ipsilesional taVNS led to a significant increase in activation in the ipsilesional visuomotor and secondary visual cortices, while simultaneously reducing visuomotor activity in the contralesional hemisphere. These results highlighted the strong influence of laterality on the effectiveness of taVNS, with ipsilesional stimulation having the most direct effect on brain activation. Huguenard et al. (2024) demonstrated the immunomodulatory effects of taVNS in patients with subarachnoid hemorrhage. This study excluded patients with SAH of traumatic etiology, patients with negative vascular imaging for aneurysm, and those receiving ongoing cancer therapy or immunosuppressive medication. taVNS significantly reduced pro-inflammatory cytokine levels in the plasma, attenuated moderate-to-severe vasospasm, and increased vessel caliber, highlighting its potential therapeutic role.

For the clinical evaluation to verify the effects of taVNS, FMA-UE was frequently used, with some studies also including assessments of the FMA lower extremity and the FMA sensory function. Motor performance outcomes, such as the Wolf Motor Function Test, timed up and go test (TUGT), Modified Barthel Index, and Berg Balance Scale scores, were measured. Only one study has used MEPs to assess cortical excitability (Wang M. H. et al., 2024). The evaluation of mental function included scales for depressive symptoms, such as the Hamilton Rating Scale for Depression and Hospital Anxiety and Depression Scale. fMRI was used to assess the effectiveness of taVNS.

### 3.4 Parkinson’s disease

#### 3.4.1 Safety

Four studies reported no adverse reactions during the trials (Fu et al., 2024; Van Midden et al., 2024b; Zhang et al., 2023; Zhang et al., 2024). Lench et al. (2023) reported that some participants who received either taVNS or sham reported adverse events. The most frequently reported adverse event in the active taVNS group was difficulty sleeping, followed by lightheadedness, fatigue, nausea, tinnitus, tooth grinding, ear fluid, anxiety, and dizziness. The most frequently reported adverse event in the sham group was lightheadedness, followed by difficulty sleeping, headache, fatigue, difficulty concentrating, and neck pain. Only a few patients present with each of these symptoms. There were no reports of pain at the stimulation site, and no serious adverse events occurred in either group (Lench et al., 2023). One study reported no adverse events (Marano et al., 2024).

#### 3.4.2 Parameters

In all six studies, the electrodes were placed in the left ear. The specific parameters varied across each study. The parameters are summarized in Tables 5a–c. van Midden et al. (2024b) proposed that 25 Hz taVNS enhanced physical function and gait in patients with PD.

**TABLE 5:** Stimulation parameters in Patient with PD.

a. Frequency	
Frequency [Hz]	Number of published manuscripts
20	3
25	3
100	2
Median: 25 Hz, IQR: 23.75 (20–43.75)	

b. Duration	
Duration [µs]	Number of published manuscripts
200	2
300	2
500	2
Median: 300 ㎲, IQR: 225 (225–450)	

c. Current intensity	
Current intensity [mA]	Number of published manuscripts
perceptual threshold	2
200% perceptual threshold	1
maximum tolerate without pain	2
not described	1

#### 3.4.3 Efficacy

In PD, all six included studies investigated taVNS as a standalone intervention. Reported benefits included improvements in gait (speed, stride length, turning) and reductions in anxiety, with no combination interventions reported. Several studies have reported improvements in gait parameters, including gait speed, stride length, and 360° turn duration (van Midden et al., 2024b; Marano et al., 2024; Zhang et al., 2024). However, Marano et al. (2024) and Lench et al. (2023) found no significant clinical changes in the UPDRS scores in their respective reports. In terms of the mental function, Zhang et al. (2023) used the Hamilton Anxiety Rating Scale to assess anxiety symptoms and concluded that taVNS could alleviate anxiety in patients with PD. Regarding neurological background, MRI studies have shown that taVNS is associated with a widespread decrease in the amplitude of LF fluctuations (ALFF) in the right hemisphere, including the superior parietal lobule, precentral gyrus, postcentral gyrus, middle occipital gyrus, and cuneus. The ALFF in the right superior parietal lobule during taVNS was negatively correlated with the UPDRS Part III (Fu et al., 2024).

To confirm the efficacy of taVNS in patients with PD, the brain neural activity and autonomic nervous system were assessed using functional near-infrared spectroscopy, fMRI, HR monitoring, and blood pressure measurements. The UPDRS and TUGT scores were used as outcome measures to assess PD symptoms.

## 4 Discussion

This review investigated taVNS, a noninvasive neuromodulation technique, focusing on three aspects, safety, efficacy, and stimulation parameters, in healthy participants, patients with stroke, and patients with PD. A total of 154 articles, including 6,485 participants, were examined. The majority of studies in healthy participants involved young to middle-aged adults, with relatively few focused on older populations. Age-related differences in neuroplasticity and autonomic regulation may influence the response to taVNS and warrant further investigation, particularly when comparing healthy adults with patients with stroke or PD. taVNS was demonstrated to be safe, with no serious adverse events reported in healthy individuals or in patients with stroke or PD. In terms of efficacy, taVNS modulates cortical excitability and autonomic nervous system activity, leading to improvements in motor and cognitive functions, as well as learning. The results were evaluated using neurophysiological indicators and task performance. However, outcome measures are insufficiently uniform, leading to differences in results and perspectives. Although commonly used parameters have been identified, they vary across studies, and many studies have emphasized their importance. Although some studies have focused on these parameters, the number of studies remains limited. It was also suggested that the effect may be influenced by the stimulus condition.

The overall quality of the evidence base is limited. Most studies had small sample sizes, many lacked blinding or sham controls, and outcome measures and stimulation parameters were heterogeneous. Reporting of adverse events was frequently incomplete. Consequently, confidence in efficacy remains low-to-moderate, whereas conclusions on safety are more consistent but constrained by potential under-reporting. In addition, a limitation of this review is that its scope excludes other indications (e.g., depression, epilepsy, gastrointestinal or cardiac disorders), which may limit generalizability. Future reviews should extend this parameter–outcome framework to additional conditions once sufficient methodologically comparable evidence becomes available. Finally, a methodological limitation of this review is that screening and data extraction were performed by a single reviewer. Although this ensured consistency in study selection and data handling, it may have increased the risk of bias compared to a double-review process. Future reviews in this field should ideally incorporate independent screening and extraction by at least two reviewers to enhance methodological rigor.

### 4.1 Safety of taVNS

taVNS activates the ipsilateral NTS to stimulate and send impulses to the heart via the efferent cervical vagus nerve, suggesting the possibility of avoiding direct and asymmetric stimulation of the vagus nerve, unlike implanted VNS (Kim et al., 2022). Previous systematic reviews and meta-analyses have noted a low incidence of arrhythmia and bradycardia, which are concerning side effects of implantable VNS. Furthermore, cardiac side effects are rare, because taVNS is currently used to treat heart failure and atrial fibrillation (Dalgleish et al., 2021). Based on these results, taVNS is considered safe.

In this review, the adverse events associated with taVNS mainly included transient ear pain, redness, itching, and other forms of discomfort. No severe adverse effects were observed. However, safety reports may be inadequate, which can be considered a limitation of the study. Most clinical studies, including those involving patients with stroke and PD, have reported the presence or absence of adverse events and their safety. Consistent with our findings, a recent systematic review of tVNS in patients with PD reported a favorable safety profile, with no serious adverse events and only mild, transient side effects such as headache, fatigue, or local discomfort (Shan et al., 2024). This further supports the view that tVNS can be safely applied in PD when appropriate stimulation parameters are used. However, 54% of the studies included in this review on healthy participants did not report the adverse events, leaving it unclear whether any occurred.

Previous reviews have stated that taVNS is well-tolerated and safe in humans at the doses tested, with no adverse events directly attributable to it. However, concerns have been raised regarding underreporting, mandatory reporting of safety and tolerability results, and the need for standardization of adverse event measurement methods. Moreover, the different taVNS parameters used in studies reporting adverse events may not allow accurate comparisons (Redgrave et al., 2018). Based on these insights, although this review partially supports previous findings on the safety of taVNS, the evidence remains insufficient for generalization. Future studies should thoroughly report side effects and adverse events as well as focus on the conditions of use, including stimulation parameters, to ensure safety.

### 4.2 Parameters of taVNS

This review found that although parameters are important for safety and efficacy, more consistent parameters and measures are required to assess them. The most frequently used frequency was 25 Hz, and the duration was 200–300 μs, which was consistently adopted. However, there are various ways to determine the current intensity, such as below the pain threshold, at the sensory threshold, twice the sensory threshold, and mild tingling. This result highlights the importance of not only maintaining consistency in the intensity value itself but also clearly defining how the intensity is set. Future studies focusing on the current intensity are necessary. Similarly, the stimulation time and duty cycle significantly vary among studies and should be considered in the context of subject burden and safety. Although most included studies applied taVNS to the left ear, partly owing to theoretical concerns regarding cardiac parasympathetic innervation of the right vagus, right-sided and bilateral approaches were also reported as safe and effective. Laterality is a crucial parameter, and although the majority of studies used left-ear stimulation for safety reasons, studies employing right-sided or bilateral stimulation similarly demonstrated safety and effects. Future research should continue to explore laterality to determine the conditions for optimal taVNS effectiveness. Parameter considerations have received attention in studies of healthy participants, and further studies aimed at clarifying stimulus parameters are expected in the future. The outcome measures were relatively consistent in basic studies including healthy individuals but varied owing to the multiple measures of autonomic assessment, such as HR, HRV, pupil size, and sAA. However, another limitation is the variability across studies, largely because of the small number of available clinical investigations. Therefore, in the present review, the variability in the assessment measures identified as challenges could be due to the selection of multiple autonomic activity measures and the small number of clinical studies. A relatively large number of assessments using the same indicators in healthy participants may facilitate the establishment of evidence in the field of taVNS research by allowing for comparisons of stimulus settings and treatment outcomes.

Future studies should report the detailed and standardized parameters and metrics used. The clinical effectiveness must be tested in consistent stimulus settings, which would allow for comparisons between taVNS studies and contribute to evidence building.

#### 4.2.1 Standardized reporting and parameter recommendations

To facilitate clinical translation, future studies should adopt standardized reporting guidelines, including complete disclosure of stimulation parameters (frequency, duration, intensity relative to pain/sensory thresholds, electrode placement, and session duration) and participant characteristics (Badran et al., 2018b; Dumoulin et al., 2021; Wang M. H. et al., 2024; Zhang et al., 2024). Based on currently available evidence, commonly applied settings, such as 25 Hz frequency, 200–300 μs duration, and sub-pain threshold intensities, may serve as starting points but should be validated and optimized across conditions. Core outcome measures, including HRV for autonomic modulation, the Fugl–Meyer Assessment or UPDRS for motor outcomes, and validated cognitive and mood scales, should be prioritized to enable comparability and evidence accumulation. Inconsistencies among studies may stem not only from methodological heterogeneity but also from biological and clinical variability. Factors such as age, sex, disease severity, and concomitant treatments may modulate vagal sensitivity and responsiveness. Study design differences—including unilateral versus bilateral stimulation, task-related versus resting-state application, and acute versus multi-session protocols—also likely contribute to divergent findings (Camargo et al., 2024; Gerges et al., 2024a; Janner et al., 2018; Sellaro et al., 2015a; Yokota et al., 2022; Szulczewski et al., 2023). Addressing these dimensions in future research will be critical to clarify mechanistic underpinnings and maximize the therapeutic potential of taVNS.

### 4.3 Efficacy of taVNS

**FIGURE 4:**
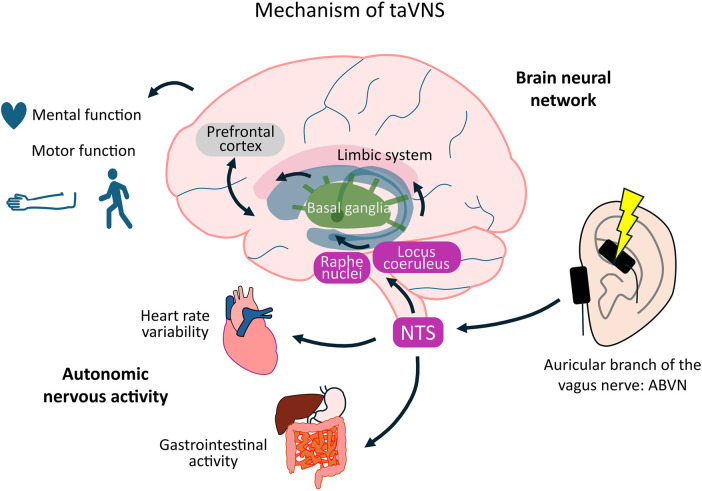
Proposed mechanism of taVNS.

The mechanism of action of taVNS is projection from the ABVN, which is an afferent fiber innervating mainly the ear, to the LC and raphe nuclei via the NTS, and modulatory effects on the cortex, limbic system, and other areas. Consequently, taVNS has shown potential for the treatment of epilepsy, depression, stroke, and PD. The vagus nerve is an important component of the parasympathetic nervous system, and taVNS increases parasympathetic activity (Kania et al., 2021). Modulation of parasympathetic activity is associated with clinical conditions, such as heart failure, inflammatory bowel disease, and chronic pain syndromes (Butt et al., 2020; Veiz et al., 2022). Mechanistically, taVNS activates afferent projections from the auricular branch of the vagus nerve to the NTS. The NTS, in turn, engages the locus coeruleus–noradrenergic and raphe nuclei–serotonergic systems, influencing widespread cortical and subcortical regions, including the prefrontal cortex, cingulate cortex, basal ganglia, and limbic areas (Ludwig et al., 2021; Huang et al., 2023). In addition, taVNS has been reported to modulate GABAergic activity in the motor cortex (Van Midden et al., 2023; Chen et al., 2024b; Keute et al., 2019a; Beste et al., 2016). Collectively, these pathways provide a neurophysiological basis for the observed effects of taVNS on cognition, motor control, and emotional regulation (Johnson and Steenbergen, 2022). This review focuses on the applications of taVNS in central nervous system diseases. These findings indicate that taVNS enhances activity in brainstem regions, such as the NTS, LC, and raphe nuclei, and in the insular cortex, thalamus, prefrontal cortex, motor cortex, cingulate cortex, amygdala, and striatum, as observed in experiments utilizing fMRI and EEG. This stimulation increases the cortical excitability and affects motor function, attention, and emotion regulation. Modulatory effects on autonomic nervous system activity have also been reported using biomarkers, such as HR, blood pressure, HRV, pupil diameter, and salivary amylase. These markers are promising in determining the effect of taVNS. These effects have also been observed in previous patient studies. Modulation of these brain regions and their functions have also been observed in healthy participants, although some reports have found no effects on HRV, pupil diameter, sAA, or memory performance. These differences indicate that consistent results have not been achieved. Thus, the present review found that the effects of taVNS were typically consistent with the mechanism of action described in previous studies; however, further studies are needed to confirm whether consistent results can be achieved (Figure 4).

In clinical reserches, in stroke patients, taVNS has been used in combination with rehabilitation and tDCS. However, because most combination studies did not implement full factorial designs or report interaction terms, current evidence supports an added benefit of taVNS when layered onto rehabilitation or tDCS, rather than demonstrating synergy *per se*. Differences in session number, stimulus dosing, and small samples further limit causal attribution. Future trials should adopt adequately powered factorial designs with matched dosing and pre-specified interaction analyses to formally adjudicate additivity vs. synergy. In patients with PD, after our search window closed, a recent meta-analysis reported no significant overall improvement in global motor symptoms with non-invasive VNS in PD, while suggesting a possible benefit for freezing of gait; these findings reinforce the need for larger, well-controlled trials with harmonized endpoints (Abouelmagd et al., 2025). Further research and discussion are required to examine the consistent efficacy of taVNS in clinical studies.

## 5 Conclusion

taVNS appears to be safe and shows promise for modulating motor, cognitive, and autonomic functions in healthy individuals, as well as in patients with stroke and PD. However, the current evidence is limited by small sample sizes, heterogeneous protocols, and short-term follow-up. Future research should prioritize long-term safety monitoring, standardization of stimulation parameters, harmonization of outcome measures, and large-scale multicenter clinical trials to generate definitive evidence for clinical application.

## Abbreviations

ABVN, auricular branch of the vagus nerve; EEG, electroencephalogram; fMRI, functional magnetic resonance imaging; HR, heart rate; HRV, heart rate variability; LC, locus coeruleus; NTS, nucleus tractus solitarius; PD, Parkinson’s disease; sAA, salivary alpha-amylase; taVNS, transcutaneous auricular vagus nerve stimulation.
