# Supplementary material for: Transcutaneous auricular vagus nerve stimulation in healthy individuals, stroke, and Parkinson’s disease: a narrative review of safety, parameters, and efficacy
Source: Front Physiol. 2025 Oct 24;16:1693907. doi: 10.3389/fphys.2025.1693907 (PMC12593472; doi:10.3389/fphys.2025.1693907)
Supplement: Supplementary file 1 [file DataSheet1.pdf]

Supplementary Table 1

Author	Sample Size	Frequency (Hz)	Pulse Width (μs)	Intensity (㎃)	Duty Cycle	Session Schedule
Alicart et al.	21	25	250	0.6	30 sec on, 30 sec off	60 min
Altinkaya et al.	14	25	250	without pain or discomfort	30 sec on, 30 sec off	approximately 90 min, in 4 locations on separate days
Antonino et al.	13	30	200	perceptual threshold	continuous	15 min
Anzolin et al.	7	100	300	detection threshold	-	5 min/session total 10min
Austelle et al.	24	25	500	200% of the perceptual threshold	2 min (no off cycle)	2 min
Badran et al.	17	25	500	200% of the perceptual threshold	30 sec on, 60 sec off 3sets	repeated 3 times over 6 min
Badran et al.	15	1, 10, 25	100, 200, 500	200% of the perceptual threshold	1 min	1 min, 9 blocks
Beste et al.	51	25	200-300	0.5	30 sec on, 30 sec off	during the task
Bömmer et al.	29	25	250	below the pain threshold	Short: 3.4 sec<br>Long: 30 sec	totally 45 min (active and sham) during the task
Borges et al.	61	25	200-300	0.5, 1.0, 1.5	30 sec on, 30 sec off	5 min
Borges et al.	32	25	200-300	the average of the 4 intensities rated as 8/10	30 sec on, 30 sec off	4 min, 2 visits for active or sham
Borgmann et al.	12	25	250	the lower sensory threshold	continued	7 min
Burger et al.	31	25	-	0.5	30 sec on, 30 sec off	Task was tested 24 hours later.
Burger et al.	58	25	250	0.5	30 sec on, 30 sec off	10 min
Burger et al.	42	25	250	0.5 or lower (when the	30 sec on, 30 sec off	during leg stimulation,

				participant feels pain at 0.5mA)		3 sessions on separate days
Burger et al.	85	25	250	0.5	30 sec on, 30 sec off	25 min
Camargo et al.	44	30	200-250	choose a comfortable intensity (0.5, 1.0, 1.5, 2.0, or 2.5 mA) below the pain threshold	continuous	60 min
Capone et al.	10	20	300	the detection threshold and below the pain threshold	repeated every 5 min for 60 min	6 min
Capone et al.	19	20	300	0.5, 1.0, 2.0, 3.0	continuous	2 min
Chen et al.	28	25	200-300	0.5	30 sec on, 30 sec off	67 min
Chen et al.	22	25	200-300	slight tingling sensation	continuous	20 min
Chen et al.	59	25	250	strong but non-painful sensation.	continuous	20 min
Chen et al.	59	25	250	strong but non-painful sensation	-	20 min
Chen L et al.	28	25	200-300	0.5	30 sec on, 30 sec off	during the task, 2 sessions (task only → task & taVNS or sham)
Cibulcova et al.	78	25	250	detection threshold (tingling sensation)	constant stimulation	4h/day, 2-week course
Clancy et al.	48	30	200	detection threshold	continuous	15 min
Colzato et al.	80	25	200-300	0.5	30 sec on, 30 sec off	15 min
Colzato et al.	32	25	200-300	0.5	30 sec on, 30 sec off	20 min, 2 sessions (active and sham), separated by 1 week

Crupper et al.	31	5 , 25	200	minimum threshold, and the onset of pain or irritation	manually turned on and off by a trained researcher	during the task
D'Agostini et al.	43	25	200, 400	0.2, 0.5, calibration intensity	5s on 20s off/block	3 min/block, 16 blocks, scheduled minimum 48 h and maximum 10 days apart
D'Agostini et al.	71	25	250	below the pain threshold	30 sec on, 30 sec off	40 min
D'Agostini et al.	66	25	250	the maximal level below pain threshold	-	50-55 min
Dalgleish et al.	28	10	300	mild tingling sensation	-	15 min
De Couck et al.	60	25	250	clear tingling or pulsating sensations without any pricking pain or unpleasant perception	30 sec on, 30 sec off	10 min
Dietrich et al.	4	25	250	below the pain threshold (between 4 and 8 mA)	30 sec on, 30 sec off	50 sec, 4 sessions
Dumoulin et al.	37	25	250	maximal, but non-painful, tingling sensation	30 sec on, 30 sec off	approximately 1 hour
Ferreira et al.	42	25	500	0.5	30 sec on, 30 sec off	20 min
Ferstl et al.	85	25	-	mild pricking	30 sec on, 30 sec off	during the task, 2 sessions (active and sham)
Finisguerra et al.	24	25	200-300	0.5	30 sec on, 30 sec off	60 min
Fischer et al.	21	25	200-300	above the detection threshold and below the pain threshold	continuous	during the task, 2 consecutive days
Forte et al.	28	25	200-300	maximally comfortable stimulation levels	30 sec on, 30 sec off	10min, 2 sessions, at the same hour and day of the week

Frokjaer et al.	18	30	250	0.1−10 mA: tingling sensation, but not painful	-	60 min, 2 sessions, separated at least 7 days
Gadeyne et al.	39	25	250	detection threshold but below pain threshold	7s on 18s off	17 min
Galvez-Garcia et al.	42	25	200-300	detection threshold	continuous in a series of 10 sec increasing and decreasing trial	during the task
Gancheva et al.	10	25	250	tingling sensation	continuous	14 min, 2 different days spaced by at least 7 days
Gauthey et al.	28	5, 20	200	detection threshold	-	10 min, wash-out: 10min
Geng et al.	27	25	250	detection threshold (5−35 mA)	continuous	10min, 2 sessions, in the morning and evening
Geng et al.	34	Study1: 20 Study2: 5, 20	Study1: 250 Study2: 50, 250	detection threshold	-	5 min
Gerges et al.	23	25	250	maximal tolerable level	30 sec on, 30 sec off	taVNS for 30 or 60 min (sham: 30 min)
Gianlorenco et al.	44	30	200-250	60/NRS (0-100)	-	30 min, 2 visits (screening and intervention)
Giraudier et al.	60	25	200-300	average of the intensities rated as 8：0.5 to 2.5mA	30 sec on, 30 sec off	23 min
Gurtubay et al.	20	20	300	below the discomfort threshold	-	7 min, 2 different days
Hatik et al.	90	10	300	detection threshold	-	20 min, 4 days protocol
Honda et al.	45	25	150	detection threshold	-	during the task

Horinouchi et al.	24	25	250	detection threshold	28 sec on, 32 sec off	30 min or continued 15 min, 3 sessions on separated days with at least a 2 day
Jackowska et al.	78	25	200-300	detection threshold	no on/off cycles	4 h/day in several time segments, 14 days
Jacobs et al.	30	8	200	5	-	17 min, 2-visit at the same time of the day, separated by 7-10 days
Janner et al.	49	pattern of 100/2 Hz	200	clearly perceptible tingling sensation but not uncomfortable or painful	-	25 min, 4 sessions, separated by at 48 hours
Johnson et al.	38	25	200-300	0.5	30 sec on, 30 sec off	approximately 1 h: applied 15 min before the tasks until their completion, 2 different days spaced by at least 7 days
Jongkees et al.	40	25	200-300	0.5	30 sec on, 30 sec off	15 min
Kaduk et al.	36	25	250	mild pricking	30 sec on, 30 sec off	30 min, 4 sessions
Kania et al.	27	10	300	mild tingling sensation	300	15 min
Keatch et al.	17	24	-	pain threshold (0.2 - 2 mA).	-	10 min, 4 sessions
Keatch et al.	17	24	-	80% of pain threshold	-	10 min/session, 4 sessions
Keute et al.	18	25	200	below pain threshold	30 sec on, 30 sec off	25 min, 2 different days, spaced by at least 2 days
Keute et al.	41	25	200	1	60 sec on 30 sec off	30 min, 2 sessions separated by at least 2 days apart, at the same daytime
Keute et al.	33	25	200	3	-	during the session, 2 sessions separated by at least 2 days apart, at the same daytime
Keute et al.	34	25	250	3	30 sec on, 30 sec off	30 min, 2 sessions (active and sham)
Konakoglu et al.	90	10	300	-	-	20 min

Konjusha et al.	45	25	200-300	0.5	30 sec on, 30 sec off	20 min, 2 sessions (active and sham)
Konjusha et al.	37	25	200-300	sensation value of 8	continuous stimulation	20 min, 2 sessions at least 1 week apart
Kozorosky et al.	10	10	300	tingling sensation	-	30 min, 2 sessions at least 1 week apart
Kraus et al.	22	8	200	noticeable (LOW), pain threshold (high)	30 sec, 1min rest	30 sec, resting period of 1 min<br>4 periods, in the fMRI
Kuhnel et al.	39	25	-	mild prickling	30 sec on, 30 sec off	60 min before the task
Laqua et al.	22	2/100 Hz bursts	200	maximal but non-painful by interviewing	-	30 min, 2 sessions
Le Roy et al.	44	25	200-300	0 to 10 "the strongest sensation imaginable"	30 sec on, 30 sec off	20 min
Llanos et al.	36	25	150	perceptual threshold	-	during the training
Lloyd et al.	29	25	200-300	rating of 9 (just below painful)	3.4 sec/trial	25 - 27 sec/block, 8 blocks, 11 trials
Lucchi et al.	40	25	-	mild pricking	30 sec on, 30 sec off	~40 min, 2 sessions, scheduled between 2 and 8 days from each other at approximately the same time of day
Machetanz et al.	13	25	100, 260, 500	below uncomfortable sensations	30 sec×3	30 sec/condition, 8 different conditions
Maharjan et al.	20	10, 80	180	no perceived pain	-	10 min
Mao et al.	27	20k	20	75% of a subject's perceptual threshold	continuous	15 min, 2 sessions at least 1 day apart
Maraver et al.	43	25	200-300	0.5	30 sec on, 30 sec off	15 min
Martina et al.	44	25	250	detection threshold and below	continuous	10 min, 2 sessions at least 2 week apart

				the pain threshold		
Mertens et al.	15	25	250	below the pain threshold	7s on 18s off	60 min
Mertens et al.	65	25	250	0.1mA below the pain threshold	30 sec on, 30 sec off	2 min, 3conditions, 30 min of wash-out
Molefi et al.	14	20	200	1	-	20 min, 2 sessions at least 1 day apart
Muller et al.	31	25	-	mild pricking (possible range 0.1-5 mA)	30 sec on, 30 sec off	10 min, in the fMRI
Müller et al.	82	25	-	below the pain threshold	30 sec on, 30 sec off	during the task, 2 sessions approximately at the same time in the morning at least 2 and up to 7 days apart
Neuser et al.	81	25	-	mild pricking	30 sec on, 30 sec off	during the task, 2 sessions (active and sham)
Ng et al.	16	20, 80	60	detection threshold and below pain threshold	-	8 visits: (I) right, canal, 80 Hz, (II) right, concha, 80 Hz, (III) left, concha, 80 Hz, (IV) left, canal, 80 Hz, (V) right, canal, 25 Hz, (VI) right, concha, 25 Hz, (VII) left, concha, 25 Hz, (VIII) left, canal, 25 Hz 10 min
Obst et al.	31	25	-	0.6	30 sec on, 30 sec off	1.9 h, 2 sessions at least 1 day apart
Peng et al.	24	20	250	detection threshold and below pain threshold	-	30 sec,4 sessions
Percin et al.	76	25	250	suprathreshold current (0.13–50 mA)	-	20 min
Petersen et al.	10	25	250	Maximal tolerated but no pain	30 sec on, 30 sec off	15 min

Phillips et al.	45	300	50	at 0.2 mA below perceptual threshold	continuous	10 min, during the video
Pihlaja et al.	25	30	250	until detected slight tingling	-	10 min, 4-different stimulation, 4min rest per each stimulation
Poppa et al.	45	25	250	without pain or significant unpleasantness	7 sec on and 18 sec off	15 min, 2 sessions (active and sham)
Rufener et al.	22	30	200	4	continuous	37 min
Rufener et al.	20	25	250	0.5	30 sec on, 30 sec off	100.5 min, 3 sessions (tVNS, transcranial random noise stimulation: tRNS, and sham), each separated by at least 3 days
Sara et al.	49	25	250	perceptual threshold	30 sec on, 30 sec off	60 min, 2 sessions at least 24 h apart
Sclocco et al.	16	25	450	percept-matching across subjects (4 to 5 on the 0-10 NRS)	-	8 min
Sclocco et al.	30	2, 10, 25, 100	300	"moderately strong, but not painful sensation," corresponding to a target score of 4-5 on the 0-10 scale	train duration of 1.5 sec	8.5 min a single MRI session
Sellaro et al.	24	25	200-300	0.5	30 sec on, 30 sec off	during the task, 2 sessions (active and sham)
Sellaro et al.	24	25	200-300	0.5	30 sec on, 30 sec off	15 min: during tasks, 2 sessions (active and sham) at least 1 day apart
Sellaro et al.	40	25	200-300	0.5	30 sec on, 30 sec off	30 min, during the tasks
Sharon et al.	24	25	200-300	rating of 8 (just below painful)	3.4 sec	5 min trialts →8 blocks, 1 block consists of 11 trials of 3.4s sec stimuli & intervals of

						25~27sec
Sinkovec et al.	15	20	100	detection threshold	-	15 min
Skora et al.	61	25	250	clearly perceptible, but not painful, sensation, within the range of 1–5 mA.	30 sec on, 30 sec off or 1 sec on, 29 sec off	4 min/session, 8 sessions, 2 visits at least 1 day apart
St Pierre et al.	24	30	100	midpoint between perception and pain threshold	0.5 sec on, 1 sec off	during the task
Steenbergen et al.	73	25	200-300	0.5	30 sec on, 30 sec off	15 min, 2 sessions (active and sham) at least 1 day apart
Steidel et al.	57	1, 25	250	below the pain threshold	30 sec on, 30 sec off	4 h
Sun et al.	104	25	500	below the light tingling threshold	30 sec on, 30 sec off	25 min, 3 sessions (online taVNS, offline taVNS, and sham)
Szeska et al.	80	25	200-300	below the pain threshold	30 sec on, 30 sec off	during the task
Szeska et al.	80	25	200-300	perceptible but below the pain threshold	30 sec on, 30 sec off	10 min
Szulczewski et al.	22	100	400	below the pain threshold	6 sec on, 4 sec off	20 min, 2 sessions (active and sham) of breathing at 6 breaths/min
Teckentrup et al.	40	25	200	pain thresholds using VAS ratings	30 sec on, 30 sec off	30 min, 2 sessions (active and sham)
Teckentrup et al.	22	25	-	pain thresholds	30 sec on, 30 sec off	30 min, consecutive days
Thakkar et al.	37	5	200	minimum threshold and two at the upper level of comfort	-	6-8 sec per sequence, during approximately 215 letter-sound pairings per session
Tobaldini et al.	13	25	200	perceptual threshold (comfortable sensation	continuous	10 min, 2 sessions (active and sham) at least 24 h apart

				without pain)		
Tona et al.	72	25	-	0.5,1.0	30 sec on, 30 sec off	75 min, 2 sessions at least 1 day apart
Toschi et al.	16	25	450	not painful sensation	-	8 min
Van et al.	31	25, 100	300	below the sensory threshold	-	10 min
Van et al.	30	100	300	0.1 mA above the perceptual threshold	continuous	30 min, 2 sessions at least 5 days apart
Veiz et al.	29	30	250	20	continuous	20 min, 2 sessions at least 1 day apart
Ventura-Bort et al.	37	25	200-300	above the detection threshold and below the pain threshold	continuous	7 min, 3-visits (active, sham, assessment)
Vertanen et al.	26	30	250	without pain	-	during the task, 4 sections, 2 of active, 2 of placebo
Villani et al.	50	25	500	below perceptual threshold	brief trains of 3 sec	during the task
Vishal et al.	35	5, 25	200	detection threshold	-	during the training
Vosseler et al.	15	25	-	tingling sensation, but no pain	30 sec on, 30 sec off	for 150 min, 2 different days with 5 to 16 days for washout
Wang et al.	58	25	200-300	below the pain threshold	30 sec on, 30 sec off	during the session: 60min, 3 sessions (pre-test, a five-session training, and a post-test), each session separates 1-2 days
Wang et al.	58	25	200-300	above detection threshold but below pain perception	30 sec on, 30 sec off	approximately 60 min, 4 groups, lasted for around 2 weeks and comprised three sessions
Warren et al.	42	25	200-300	0.5	30 sec on, 30 sec off	minimum of 15 min before the task, 2 sessions (active and sham)
Warren et al.	61	25	200-300	0.5	30 sec on, 30 sec off	20 min, 2 sessions (active and sham) at least 1 day apart

Wienke et al.	29	30	200	no longer reported discomfort	-	during the task
Yakunina et al.	37	25	500	0.1 mA weaker than the pain threshold	-	6 min
Yokota et al.	20	100	250	3	-	2 min, 2 sessions (active and sham) at least 1 day apart
Yokota et al.	20	100	250	3	-	2 min, 2 sessions (active and sham) at least 1 day apart
Zhao et al.	119	25	500	detection threshold	30 sec on, 30 sec off	30 min, 3 visits for 2 experiences
Zhu et al.	82	25	500	detection threshold but not generating any discomfort	30 sec on, 30 sec off	15 min with fNIRS, 15 min with task and fNIRS
Zhu S et al.	49	25	500	tingling but not painful	30 sec on, 30 sec off	30 min, 2 sessions (active and sham) at least 1 day apart

Supplementary Table 1: Parameters of taVNS in healthy participants

Supplementary Table 2

Author	Sample Size	Frequency (Hz)	Pulse Width (μs)	Intensity (㎃)	Duty Cycle	Session Schedule
Baig et al.	12	25	100	maximally tolerated by the participant	-	during the repetitive task
Capone et al.	14	20	300	above the detection threshold and below the pain threshold	repeated every 5 min for 60 min	60 min, 10 days
Chang et al.	36	30	300	just below the pain threshold	-	60 min, 3/week, 3 weeks
Huguenard et al.	40	20	250	0.4	-	20 min, twice/day, during in ICU
Li et al.	60	20	300	adjusted according to the tolerance of each patient	30 seconds, repeated every 5 minutes	20 min, 5 times /week, 4 week
Liu et al.	80	20	300	- (average: 1.82 ± 0.4 mA)	30 sec on, 5 min intervals	45 min, 28 consecutive days
Peng et al.	20	25	500	200% Perceptual Threshold	30 sec on, 30sec off	8 min, 4 sets
Wang et al.	169	25	300	lowest level until the patient experienced discomfort	30 sec on, 30 sec off	30 min/day, 5 days/week, 4 week
Wang et al.	40	25	500	tolerable level	30 sec on, 30sec off	5 days/week for 4 weeks

Supplementary Table 2: Parameters of taVNS in stroke patients

Supplementary Table 3

Author	Sample Size	Frequency (Hz)	Pulse Width(μs)	Intensity (㎃)	Duty Cycle	Session Schedule
Van et al.	30	25, 100, sham	300	perceptual threshold	-	3 stimulation conditions during gait assessment
Fu et al.	47	20, 100	200	perceptual threshold	on–off cycle stimulation of 20/100 Hz	2 sessions (active and sham) each 8 min
Marano et al.	10	25	300	-	120 sec on, 60 sec off 4 trains	120 sec on, 60 sec off, 4 trains
Lench et al.	30	25	500	200% perceptual threshold	60 sec on30 sec off	60 min, 10 days
Zhang et al.	60	20	200	Below pain threshold	20 Hz for 7 sec, and 4 Hz for 3 sec	30 min, twice a day, 14 days
Zhang et al.	36	20	500	Below pain threshold	60 sec on, 10 sec off	30 min, 2 sessions/day, 7 days

Supplementary Table 3: Parameters of taVNS in PD patients
